# The effect of aqueous extract of *Rosa damascena* on formaldehyde-induced toxicity in mice testes

**DOI:** 10.1080/13880209.2017.1413663

**Published:** 2017-12-12

**Authors:** Majid Askaripour, Azam Hasanpour, Foruzan Hosseini, Mojgan Moshrefi, Gholamreza Moshtaghi, Mohammad Hasannejad, Soodeh Rajabi, Seyed Noureddin Nematollahi-Mahani

**Affiliations:** a Physiology Research Center and Department of Physiology, Institute of Neuropharmacology, Kerman University of Medical Sciences, Kerman, Iran;; b Department of Anatomy, Kerman University of Medical Sciences, Kerman, Iran;; c Department of Physiology, Kurdistan University of Medical Sciences, Sanandaj, Iran;; d Research and Clinical Center for Infertility, Yazd Reproductive Institute, Shahid Sadoughi University of Medical Science, Yazd, Iran;; e Department of Biochemistry, Afzalipour School of Medicine, Kerman University of Medical Science, Kerman, Iran;; f Department of Clinical Biochemistry, School of Pharmacy, Isfahan University of Medical Sciences, Isfahan, Iran;; g Department of Anatomy, Afzalipour School of Medicine, Kerman University of Medical Sciences, Kerman, Iran

**Keywords:** Formaldehyde toxicity, oxidative stress, antioxidant

## Abstract

**Context:***Rosa damascena* L. (Rosaceae) (RD) essential oil and extracts are commonly used as a flavour in herbal medicine which increase libido. Previous studies have shown inhalation of RD flower’s oil increases libido and causes protective effects in formaldehyde (FA)-induced testicular damage.

**Objective:** The protective effects of aqueous extract of RD on the male reproductive system of mice were examined following FA-induced damage.

**Materials and methods:** Forty-eight adult NMRI male mice were randomly assigned to six groups (*n* = 8): control (normal saline, 10 mg/kg); RD40 (40 mg/kg, p.o.); FA treated (10 mg/kg of 10%, i.p.) and FA + RD treated at 10, 20 and 40 mg/kg (FA + RD10), (FA + RD20) and (FA + RD40), respectively, for 40 days. At the end of treatment regimes, serum testosterone (T) level and the reproductive activity, viz. body/organ weights, testicular structure and sperm characteristics were studied.

**Results:** Formaldehyde administration significantly decreased serum T level (*p* < 0.001), testicular weight/volume, tubular diameter and sperm characteristics compared to the control group (*p* < 0.05). RD (40 mg/kg) administration in FA-treated mice significantly improved serum T level, testicular weight/histological structure, tubular diameter, Leydig cell number and epididymal sperm characteristics in comparison to its lower doses and the control group (*p* < 0.05).

**Discussion and conclusions:** We may conclude that RD flower extract can withstand effects of FA in the male reproductive system of mice possibly due to its antioxidative properties.

## Introduction

Herbal medicine has been used as a reliable source of remedies for a long time. Several plants have been recommended for the treatment of various diseases, including those that affect the reproductive system (Rivera et al. 2013). *Rosa damascena* L. (Rosaceae) (RD), commonly known as Damask rose (Kaul et al. 2000), is known as Gole Mohammadi in Iran. It is one of the most important species of Rosaceae family commonly used to increase libido as recommended in Iranian traditional medicine (Boskabady et al. 2011). Various components and substances have been isolated from the petals, hips and flowers of RD, including anthocyanins, flavonoids, glycosides, inorganic acids, fatty oil and terpenes (Kaul et al. 2000; Schiber et al. 2005). RD has also been utilized for the treatment of various medical conditions such as Alzheimer’s disease (Esfandiary et al. 2014), cardiovascular disorders (Kwon et al. 2010) and diabetes mellitus (Gholamhoseinian et al. 2009). Formaldehyde (FA) is widely used in different applications, including cosmetics, adhesives and healthcare products (World Health Organization [WHO] 2006). It is also widely used in histology, pathology and anatomy laboratories. Previous studies have shown that FA has destructive effects on the respiratory, digestive and the central nervous systems (Kriebel et al. 2001; Sarsilmaz et al. 2007). It also affects the reproductive system, it may cause infertility through testicular damage and decrease the levels of testosterone as well as sperm quality and concentration (Odeigah 1997; Sarsilmaz and Ozen 2000; Zeng et al. 2003; Wang et al. 2006; Xing et al. 2007). Intraperitoneal (i.p.) administration of FA or its inhalation has been shown to cause destructive changes in the seminiferous epithelium of mouse and rat, resulting in decreased number of spermatozoa and their motility (Oka et al. 1998; Tang et al. 2003; Tootian et al. 2007; Kose et al. 2012). However, inhalation of RD flowers oil has been shown to cause protective effects in FA-induced testicular damage and sperm parameters (Kose et al. 2012). Therefore, the present study was conducted to evaluate the protective effects of flower extract of RD in FA-induced reproductive toxicity in male mice.

## Materials and methods

### Extract preparation

Fresh *Rosa damascena* petals and leaflets were collected from Kashan, Iran, in the month of April 2014. It was authenticated by Zahra Mahdavi and Fariba Sharififar botanists, in the Herbal and Traditional Medicine Research Center, Kerman, Iran: a specimen (voucher no.: KF1362) was deposited in the Herbarium of the Faculty of Pharmacy and Pharmaceutical Sciences, Kerman University of Medical Sciences, Kerman, Iran. The plant material was washed, shade-dried in the air and powdered. The aqueous extract was prepared by adding 60 g of crude materials to 300 mL distilled water and lasted 48 h at 4 °C. The extract was filtered to isolate the harsh materials, dried and condensed with rotary evaporation high vacuum drying machine (Heidolgh, Schwabach, Germany) at 50 °C and freeze dryer (Eyela, Tokyo, Japan), respectively, to gain the solid extract (yield: 20% relative to dry plant). The dried extract was stored at −20 °C until it was used in experimental work. Various doses of 10, 20 and 40 mg/kg RD were prepared freshly by dissolving the extract in distilled water and stored at 4 °C (Himesh et al. 2012). Due to the length of the experiment, the extraction process was repeated several times.

### Animals

In the experimental design of study, 48 adult NMRI male mice (25–30 g each) of strain were obtained from the Kerman University of Medical Sciences, Kerman, Iran, after the institutional ethical review board’s permission (approval number 69-1780). Animals were housed under standard conditions of 12 h light/dark cycle and free access to water and standard laboratory mice chew (Borjsanat Company of Laboratory Rodent Diet, Tehran, Iran).

### Experimental design

As the maximum period of spermatogenesis in mice was reported to be 40 days (Cheng 2009), the experiments continued for 40 days. The animals were randomly allocated (*n* = 8) into control, which received normal saline (10 mg/kg); FA, which was given 10 mg/kg a single daily i.p. dose of 10% FA (Tootian et al. 2007; Tajaddini et al. 2014); three groups, which received 10, 20 and 40 mg/kg/day RD extract P.O. following FA administration (FA + RD10, FA + RD20 and FA + RD40) and a RD (RD40). A group, which received 40 mg/kg P.O. The median lethal dose (LD_50_) value was 16 mg/kg for FA and median effective dose (ED_50_) value was 8.2 mg/kg for RD.

### Testosterone hormone measurement

On day 41, the animals were anesthetized by ether inhalation, blood was aspirated from the left ventricle and serum was collected by centrifugation at 2000 rpm for 10 min and supernatant stored at −20 °C. The serum level of testosterone was measured by enzyme-linked immuno-absorbent assay (ELISA) kit (Diaplus, USA; Diagnostic System Laboratories Inc., STAT FAX, Webster, TX) as recommended by the manufacturer. animals were autopsied and the male reproductive organs (viz. testis and epididymis) were dissected out surgically, weighed and fixed in 10% neutral buffered formalin for histology purpose.

### Testis morphology/histology

Testis weights and volume were measured from control and treated groups. A standard calliper was used to measure testis width and length. The testes were fixed with 10% neutral buffered formalin for routine histological preparations of slides by haematoxylin–eosin (H–E) staining method. The testes sections (5 μm) were examined under an optical microscope (Olympus, Tokyo, Japan) at 200× magnification and microphotographed with a digital camera (DP71, Olympus, Tokyo, Japan). The thickness of the germinal layer of seminiferous tubules, diameter of seminiferous tubules, number and morphology of Leydig cells were evaluated by Image analysis Software (Olympus Soft Imaging Solutions GmbH, Münster, Germany).

### Sperm analysis

The caudal part of the left epididymis was taken out and placed in 1 mL human tubal fluid (HTF) supplemented with 4 mg/mL BSA and cut into several fragments to allow the spermatozoa to come out from the reproductive ducts. Samples were incubated for 15 min at 37 °C and the following parameters (e.g., sperm number, motility, viability and morphology) were studied in control and treated group of animals. Sperm number was assessed by an improved Neubauer hemocytometer technique (CNMEDITECH, Jiangsu, China); sperm suspensions were diluted 1:20 in a diluting solution (50 g/L Na_2_HCO_3_ and 1% FA; Merck, Kenilworth, NJ) in distilled water. The diluted samples were put into the counting chamber and a number of sperms were counted under a light microscope (Nikon Ts100, Tokyo, Japan) and expressed as sperm number/mL. Sperm motility was analysed by counting 200 motile (sperm with slight movement without forward progression were also considered motile) and immotile sperm in at least five microscopic fields (400×). Sperm viability was determined by the eosin (EO) dye exclusion test. For the EO test, the sperm suspension was mixed thoroughly and 10 µL of the suspension was mixed with 10 µL of EO-nigrosin dye. A thin smear was prepared after 1 min and the number of viable sperms was determined out of 200 sperms in at least 10 microscopic fields (400×). The live spermatozoa remained unstained and the dead ones were stained red. For sperm morphology assessment, a smear of sperm sample was fixed with formalin, dehydrated by graded alcohols in ascending order and stained with EO. After preparation, the slides were examined under a light microscope at 400× magnification. For each sample, 300 sperm cells were examined on each slide and the number of morphologically normal sperm was assessed. To assay sperm abnormality, smears were prepared from the sperm suspension (10 μL) and stained with the Papanicolaou method.

### Statistical analysis

The results were reported as mean ± SD. The sperm motility and viability data were reported as a percentage. For statistical analysis, one-way ANOVA was used with *post hoc* Tukey test and chi-square analysis where applicable. *p* < 0.05 was considered statistically significant and statistical analysis was performed using SPSS20 (SPSS Inc., Chicago, IL).

## Results

### Testosterone hormone level

**Figure 1.**
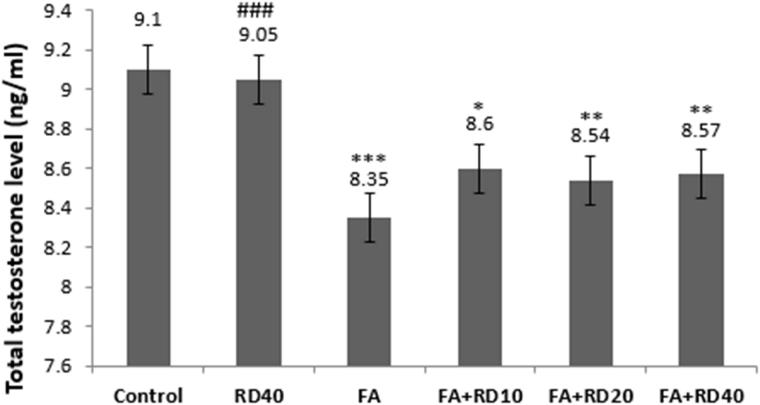
Testosterone levels of all groups in the male rat. *Significant differences vs. control group (**p < 0.01, ***p < 0.001). #Significant differences vs. FA group (###*p* < 0.001). Data are expressed as mean ± SD. RD: *Rosa damascena*; FA: formaldehyde.

Testosterone level was comparable between control and RD (40 mg/kg)-treated animals for 40 days. But, it significantly decreased (*p* < 0.001) in FA-treated group as compared to control. RD treatment at 10, 20 and 40 mg/kg to FA-injected animals significantly increased the testosterone level as compared to FA treated group, but it was highly significant in RD40 as compared to lower doses (*p* < 0.001) (Figure 1).

### Testis parameters/histology

Treatment of the rats with FA significantly (*p* < 0.001) decreased testes parameters including weight, volume, length and width, compared to control group. While treatment of FA-treated animals with different doses of RD significantly increased these parameters. This was in a dose-dependent manner when compared with the FA-treated animals (*p* < 0.05). However, treatment of RD at 40 mg/kg RD did not show any improvement in these parameters as compared to control group (*p* > 0.05) (Table 1).

**Table 1.** Testis weight, testis volume, testis length and testis width in different groups (mean ± SD, *n* = 8).

Group/parameters	Weight (mg)	Volume (mm^3^)	Length (mm)	Width (mm)
Control	113.8 ± 1.1	118.6 ± 4.69	7.9 ± 0.2	6.052 ± 0.1
RD40	112 ± 1.76###	118.6 ± 3.76###	7.7 ± 0.2##	5.88 ± 0.2###
FA	99.8 ± 2.26***	96.4 ± 0.93***	6.9 ± 0.01**	4.93 ± 0.1***
FA + RD10	106 ± 1.76**,#	102.6 ± 3.76**	7.5 ± 0.2#	5.072 ± 0.04***
FA + RD20	107.8 ± 2.8*,##	101.4 ± 2.11***	7.2 ± 0.1**	5.47 ± 0.2**,#,$
FA + RD40	111 ± 1.43###	105 ± 2.76**	7.8 ± 2.0##	5.6 ± 0.1*,##,$$

RD: *Rosa damascena*; FA: formaldehyde.

Data are expressed as mean ± SD.

* Significant differences vs. control group (*p < 0.05, **p < 0.01, ***p < 0.001).

#Significant differences vs. FA group (#*p* < 0.05, ##*p* < 0.01, ###*p* < 0.001).

^$^Significant difference vs. FA + RD10 group (^$^*p* < 0.05, ^$$^*p* < 0.01).

**Figure 2.**
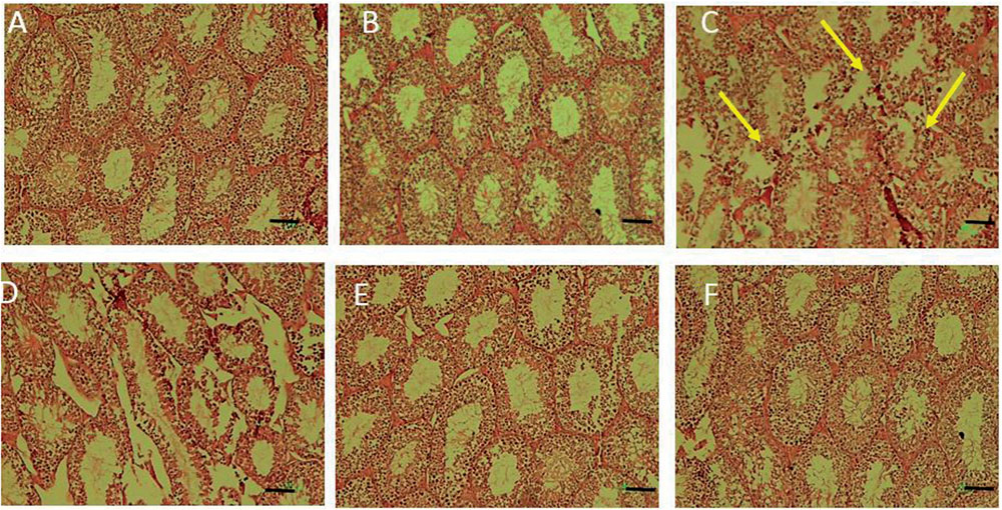
Histological examinations of tubules in the testis of (A) control, (B) sham (RD40), (C) FA, (D) RD10 + FA, (E) RD20 + FA and (F) RD40 + FA groups. Abnormal and barren testicular seminiferous without spermatozoa were observed in germinal epithelium of FA group. In FA and RD + FA groups, the diameters of testicular seminiferous and epithelial tubules significantly decreased compared to the control group. However, in the ‘FA + RD’ groups, the diameters of seminiferous and epithelial tubules increased compared to the FA group. Scale bar =100 µm.

Histological examinations of the testes from control and RD40-treated groups of mice showed normal seminiferous epithelium exhibiting all germ cell types, e.g., spermatogonia, spermatocytes, spermatids and spermatozoa (*p* > 0.05). However, mice treated with FA, caused destructive changes in the seminiferous epithelium exhibiting degeneration of the germ and Leydig cells, significant decreases in diameter of seminiferous tubules, epithelial thickness and Leydig cell number as compared to control group of mice (*p* < 0.05). In animals treated with FA + RD at 10 (FA + RD10), 20 (FA + RD20) and 40 (FA + RD40) mg/kg showed significant increase in germ and Leydig cells number, testicular tubular diameter and thickness as compared to FA-treated mice (*p* < 0.05) (Table 2; Figure 2(A–F)).

**Table 2.** Number of Leydig cell, diameters and thickness of seminiferous tubules in different groups (mean ± SD, *n* = 8).

Group/parameters	Diameter ofseminiferous tubules (µm)	Thickness ofseminiferous tubules (µm)	Number of the Leydig cells(in an interstitial area, µm)
Control	129.5 ± 1.5	34.5 ± 0.77	38.6 ± 0.68
RD40	129.2 ± 1.6###	34.1 ± 0.62###	38.6 ± 1.3###
FA	110.4 ± 1.7***	29.7 ± 0.58***	30.8 ± 0.8***
FA + RD10	111.6 ± 1.6***	29.5 ± 0.4***	32.6 ± 0.68***
FA + RD20	113.7 ± 1.2 ***	30 ± 0.32***	33.6 ± 0.75**,#
FA + RD40	113.3 ± 1.2***	30.32 ± 0.46***	33.2 ± 0.57**,#

RD: *Rosa damascena*; FA: formaldehyde.

Data are expressed as mean ± SD.

* Significant differences vs. control group (**p < 0.01, ***p < 0.001).

#Significant differences vs. FA group (#*p* < 0.05, ###*p* < 0.001).

### Epididymal sperm parameters

**Figure 3.**
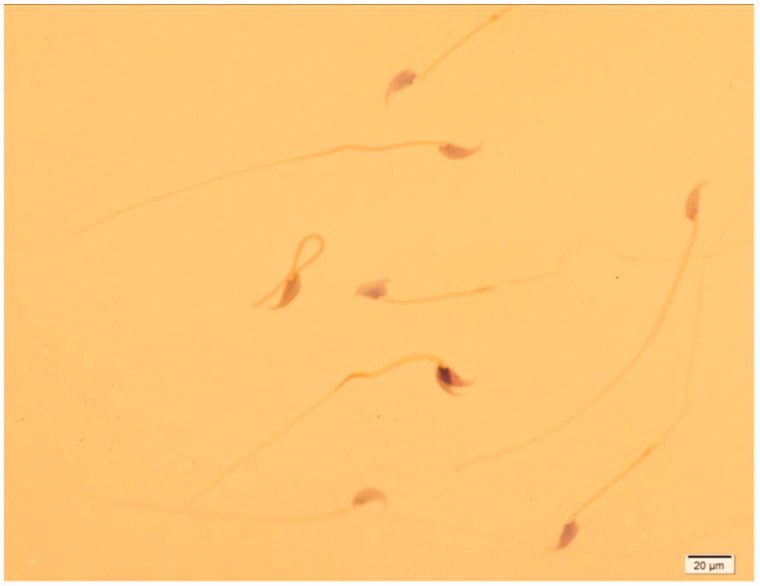
Different types of sperm shape abnormality in FA group vs. control group. Sperm suspension was smeared onto glass slides and stained using the method of Papanicolaou. Spermatozoa were counted and categorized as normal, head abnormal, neck abnormal and tail abnormal spermatozoa, ×100 magnification.

Treatment of animals with 40 mg/kg RD did not show any significant changes in sperm parameters including its number, motility, viability and rate of normal sperms as compared to control group (*p* > 0.05). The exposure of animals with FA caused a significant decrease in these sperm parameters as compared to controls (*p* < 0.001). However, administration of RD at 40 mg/kg dose for 40 days to the FA-treated animals (FA + RD40) significantly increased these sperm parameters than that of its lower doses (FA + RD10 and FA + RD20) (*p* < 0.05). However, none of the RD doses could improve sperm parameters in FA-treated mice to the level of control group. The sperms showed abnormality in the head, neck and tail regions in mice exposed to FA as compared to control group (Table 3; Figure 3).

**Table 3.** Sperm number, sperm motility, sperm viability and rate of normal sperm of all groups (mean ± SD, *n* = 8).

Group/parameters	Number (number/mL−1)	Motility (%)	Viability (%)	Rate of normal sperm (%)
Control	240.70 ± 19.7	86.8 ± 1.49	81 ± 0.84	88.8 ± 1.65
RD40	234.20 ± 14.81###	87 ± 1.81###	79.6 ± 1.47###	87.4 ± 1.4###
FA	84.46 ± 7.94***	49.6 ± 1.96***	60.6 ± 3.1***	62.4 ± 3.4***
FA + RD10	134.88 ± 19.97***,#	68.4 ± 3.38***,###	73.6 ± 2.13*,###	69.2 ± 1.74***,#
FA + RD20	133.27 ± 15.6***	70.6 ± 3.69***,###	72.6 ± 1.5**,###	72.6 ± 2.46***,##
FA + RD40	174.16 ± 21.55*,##	72.6 ± 3.17**,###	77.8 ± 1.7###	78 ± 2.64**,###,$

RD: *Rosa damascena*; FA: formaldehyde.

Data are expressed as mean ± SD.

* Significant differences vs. control group (*p < 0.05, **p < 0.01, ***p < 0.001).

#Significant differences vs. FA group (#*p* < 0.05, ##*p* < 0.01, ###*p* < 0.001).

$Significant difference vs. FA + RD10 group ($*p* < 0.05).

## Discussion

The present study showed that testis parameters including weight and volume, seminiferous tubules diameter, germinal epithelium thickness and the number of Leydig cells as well as epididymal sperm parameters (sperm number, motility and viability) were altered following i.p. injection of FA as compared to controls. P.O. administration of RD with different doses could partially revert these effects caused by FA-injection to some extent, where 40 mg/kg dose was the most effective in increasing testicular and epididymal aspects. Previous studies with FA administration for a period of five days to 18 weeks have also shown significant changes in testis structure, sex hormone profile and sperm parameters (Oka et al. 1998; Tang et al. 2003; Zhou et al. 2006a; Golalipour et al. 2007). Furthermore, inhalation of FA (Zhou et al. 2006a, 2006b) also decreases the sperm motility, viability and number in experimental animals. Our study with 10 mg/kg i.p. administration of FA for 40 days also indicates testicular damage and affects sperms number, motility and viability. Reported studies have also suggested that i.p. FA administration may cause atrophy and degeneration of seminiferous tubules which leads to reductions in sperm number (Tang et al. 2003) by increasing the reactive oxygen species (ROS) production in many tissues including reproductive organs (Gules and Eren 2010). The excessive free radicals production in testis following FA administration may increase germ cell apoptosis which inhibits the activity of spermatozoa (Ozen et al. 2005; Zhou et al. 2006a). Even *in vitro* exposure of semen to FA inhibits sperm motility and viability (Oka et al. 1998). Our findings confirmed the inhibition of spermatogenesis and sperm characteristics in FA-treated mice. The mechanism by which FA affects the germinal epithelium and sperm profile has been elucidated to some extent. Excessive amounts of ROS, which are generated in suboptimal conditions, are linked with lipid peroxidation of the sperm outer membrane, which may lead to loss of motility (Urata et al. 2001). The changes inducing peroxidation of sperm membrane components result in reduced enzymatic activity of Na/K-ATPase (as an ion pump involved in the movement) which ultimately declines sperm motility (Woo et al. 2000). According to Tramer et al. (1998), the excessive ROS may alter lipid peroxidation of sperm cell membranes, damaging the mid-piece, axonemal structure or disrupting the capacitation and acrosomal reaction, which finally results in infertility. Abnormal sperm may propose some damage in proteins that are involved in the movements or flagellar/ciliary beating of spermatozoa (Bilaspuri and Bansal 2008). *Rosa damascena* is an antioxidant which could consume free oxygen radicals and stabilize the cell membranes (Tatke et al. 2015). The testicular spermatogenesis, the process of sperm formation is dependent on FSH and testosterone and disruption in biosynthesis of these hormones leads to inhibition of testicular spermatogenesis, sperm formation and Leydig cells morphology and accessory organs function. The atrophy of testicular tubules, degradation of seminiferous epithelial cells and changes in Leydig cell number have been shown to be associated with a decreased testicular antioxidant system following FA administration (Zhou et al. 2006a, 2006b). Reported studies have also demonstrated that i.p. administration or inhalation of FA, leads to a significant reduction in the serum testosterone levels, sperm motility and it damages the Leydig cells (Ozen et al. 2005; Zhou et al. 2006b; Kose et al. 2012). Joensen et al. (2008) demonstrated a direct correlation between sperm motility and testosterone production in Leydig cells. Our results obtained in mice also show similarity with the previous studies indicating inhibition of serum testosterone level and damage to the Leydig cells by FA. Several studies have attributed these effects to the oxidative damage caused by FA (Zararsız et al. 2004; Ozen et al. 2008). Thus, the changes in the testes determined in our study may be due to the oxidative damage caused by FA.

However, RD treatment had more likely protective effects on testicular spermatogenesis and epididymal spermatozoa in FA-treated mice which concur with previous findings (Zhou et al. 2006b; Kose et al. 2012; Tajaddini et al. 2014). The preliminary phytochemical tests revealed the presence of alkaloids, flavonoids, phenols, carbohydrate, saponins, sterols and tannins in aqueous extract of RD (Himesh et al. 2012). Flavonoids and phenolic acids are the major class of phenolics, widely distributed in rose petal (Himesh et al. 2012; Memariani et al. 2015). The main phenolics responsible for antioxidant properties of RD are quercetin and gallic acid, mostly causes the antioxidant properties in rose petal (Himesh et al. 2012; Memariani et al. 2015). Disorders in testes associated with oxidative damage were reported to improve with antioxidant therapy (Zararsız et al. 2004; Ozen et al. 2008). In our study, the significant improvement in the serum testosterone level, histopathological changes in the testes and sperm parameters by RD extract in FA-treated mice may be attributed to its antioxidant effect. However, long-term effects of RD at higher doses need to be investigated further.

## Conclusions

The present study shows that FA administration damages testicular structure exhibiting reduction in testicular tubular diameter, Leydig cell number, epididymal sperm profile and serum testosterone level. Oral administration of RD to FA-treated mice showed protective effects exhibiting improvements in these parameters, which might be attributed to its antioxidative properties. Further studies are needed to justify the use of this cost-effective and easily accessible agent in other species and similar pathological situations.
